# Exploring the efficacy of subwavelength gratings as short-wavelength infrared filters

**DOI:** 10.1186/s11671-024-04045-1

**Published:** 2024-06-17

**Authors:** Hezhuang Liu, Yixuan Huang, Jiang Wu

**Affiliations:** 1 Institute of Fundamental and Frontier Sciences, University of Electronic Science and Technology of China, Chengdu, 610054 China; 2 The State Key Laboratory of Electronic Thin Films and Integrated Devices, University of Electronic Science and Technology of China, Chengdu, 610054 China

**Keywords:** Subwavelength gratings, SWIR, Narrowband filters, Spectral sensing

## Abstract

Advancements in nanofabrication technology have greatly facilitated research on nanostructures and their associated properties. Among these structures, subwavelength components have emerged as promising candidates for ultra-compact optical elements, can potentially supplant conventional optical components and enable the realization of compact and efficient optical devices. Spectral analysis within the infrared spectrum offers a wealth of information for monitoring crop health, industrial processes, and target identification. However, conventional spectrometers are typically bulky and expensive, driving an increasing demand for cost-effective spectral sensors. Here we investigate three distinct subwavelength grating structures designed to function as narrowband filters within the short-wavelength infrared (SWIR) range. Through simple adjustments to the period of grating strips, these filters selectively transmit light across a wide wavelength range from 1100 to 1700 nm with transmission exceeding 70% and full width at half maximum (FWHM) down to 6 nm. Based on a simple design, the results present great potential of subwavelength grating filters for multiband integration and developing ultra-compact spectral sensors.

## Introduction

Spectral sensing, whether deployed in military [1], industrial [2], or agricultural contexts [3, 4], has assumed a paramount role due to its robust compositional analysis capabilities. Conventional spectrometers typically use separated optical components as dispersive elements, suffering from complex configurations and bulky systems, thereby constraining their utility, particularly for aerospace applications and in emerging portable devices. In this context, subwavelength optical structures have attracted much interest thanks to their planarity and effective light manipulation capabilities, holding promise for integration into compact systems, smartphones, or spectral imaging devices [5–8]. In the past decades, subwavelength structures have been extensively investigated and has facilitated the advancement of integrated photonics, focusing on the miniaturization of photonic systems [9]. Extensive research efforts have been devoted to narrowband filters, utilizing techniques such as Fabry-Pérot resonators [10], combinatorial deposited thin films [11], dielectric metasurfaces [12, 13], guided-mode resonance (GMR) filters [14, 15], linear variable filters [16] and subwavelength gratings [17]. To achieve spectral measurements, simultaneous multiband measurements are desirable, prompting the integration of multiband filter arrays onto a single chip. By integrating filter arrays with detector arrays offers an approach to realize real-time spectral imaging [10]. Among these efforts, subwavelength grating structures stand out for their facile light manipulation and tunability through grating period variation. Notably, subwavelength gratings have been harnessed to realize polarizers [18], color reflectors [19], narrowband filters [20–24]. For transmission applications, color filters leveraging subwavelength metal–insulator–metal (MIM) stack gratings have been reported [20]. By introducing metal gratings into a GMR structure, the subwavelength structure has demonstrated competitive near-infrared narrowband filtering capabilities for spectroscopy applications [22]. This underscores the significant potential of subwavelength gratings for fabricating large-scale filter arrays and integrating them with detector arrays, particularly for spectroscopy applications in the SWIR waveband. Nevertheless, challenges persist regarding their operational spectral range and the occurrence of sidebands, necessitating the development of filters with enhanced performance characteristics.

In this work, we investigate three different types of subwavelength grating configurations incorporating with dielectric structures for their filtering efficacy in the SWIR waveband. While filters based on plasmonic GMR (pGMR) structures work across the wavelength range of 1100 to 1700 nm, they suffer from significant performance degradation due to sidebands observed at shorter wavelengths. By comparison, MIM-stack gratings exhibit comparable center wavelength transmission to pGMR filters and mitigation on sidebands. MI-stack gratings exhibit competitive narrowband filtering capability across the target waveband, with significant sideband suppression and efficient transmission of around 80%. MI-stack gratings demonstrate superior narrowband transmission characteristics within the SWIR waveband, holding considerable promise for spectral analysis applications and integration with SWIR imagers.

## Results and discussion

**Fig. 1:**
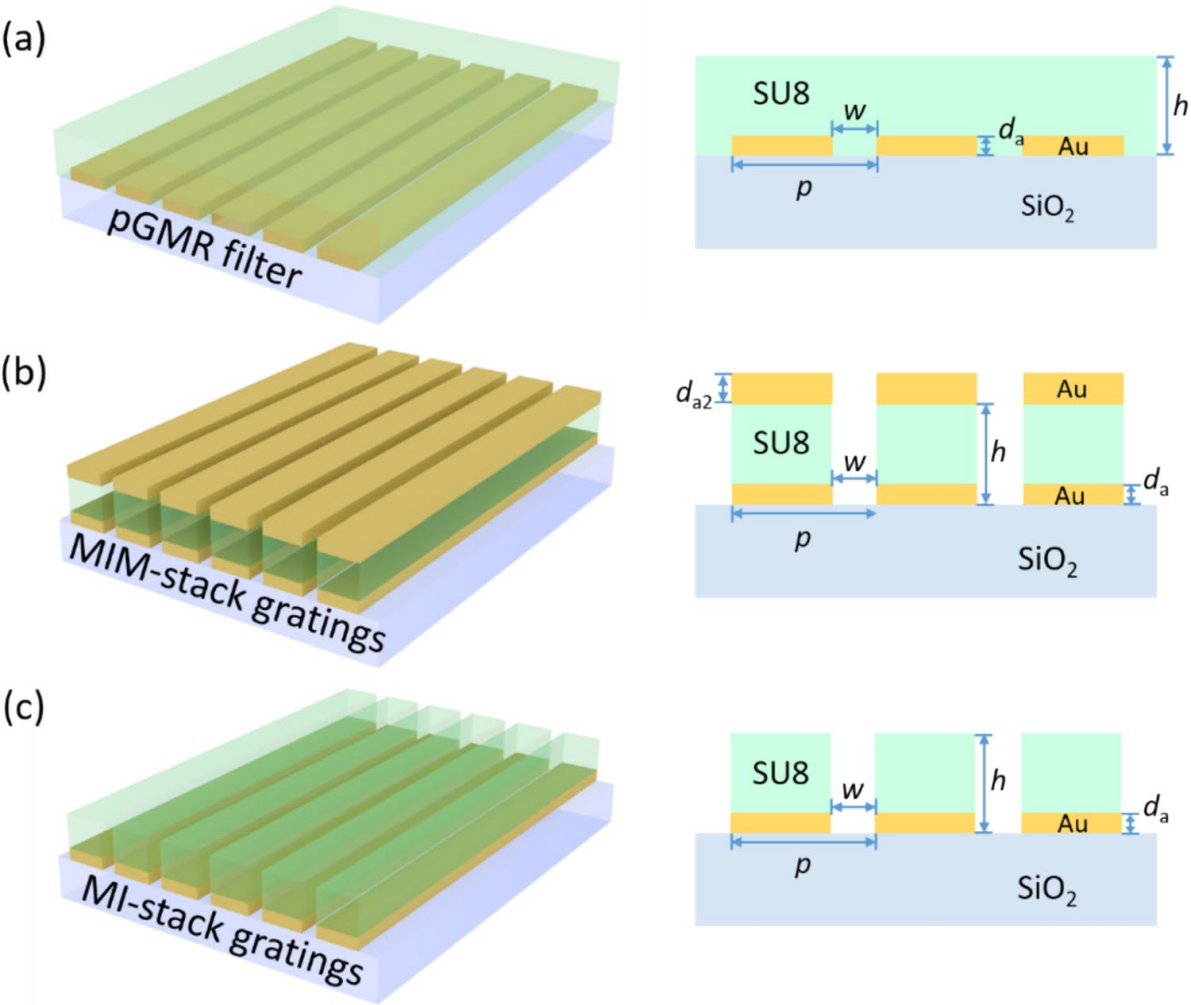
Schematic diagrams of the subwavelength grating structures under investigation. **a** pGMR filter, **b** MIM-stack gratings, and **c** MI-stack gratings

Figure 1 depicts the schematic diagrams of the investigated subwavelength gratings incorporating with diverse dielectric components to function as pGMR filters (Fig. 1a), MIM-stack grating filters (Fig. 1b), and MI-stack grating filters (Fig. 1c). These structures are designed on a quartz substrate possessing a refractive index of approximately *n*_1_ = 1.45 within the SWIR range. A layer of gold grating arrays is first placed on the quartz substrate, subsequently coated with a dielectric SU8 layer (*n*_2_ = 1.56), either forming a continuous thin film or discrete stack gratings. The relevant geometric parameters include the grating period (*p*), slit width (*w*), thickness of Au layers (*d*_a_), and the thickness of the stacks (*h*). We investigate the transmission properties of the subwavelength grating structures within the SWIR range spanning from 1100 to 1700 nm. Since the Au gratings will reflect TE-polarized light like wire-grid polarizers, the incident broadband plane wave applied is TM-polarized and illuminates normally from the substrate side. The bottom Au gratings selectively couple incident light into the dielectric layer, which then couples with guided-mode resonance or undergoes a photon-plasmon-photon conversion process, thereby yielding a narrowband transmission feature at the phase matching condition.

**Fig. 2:**
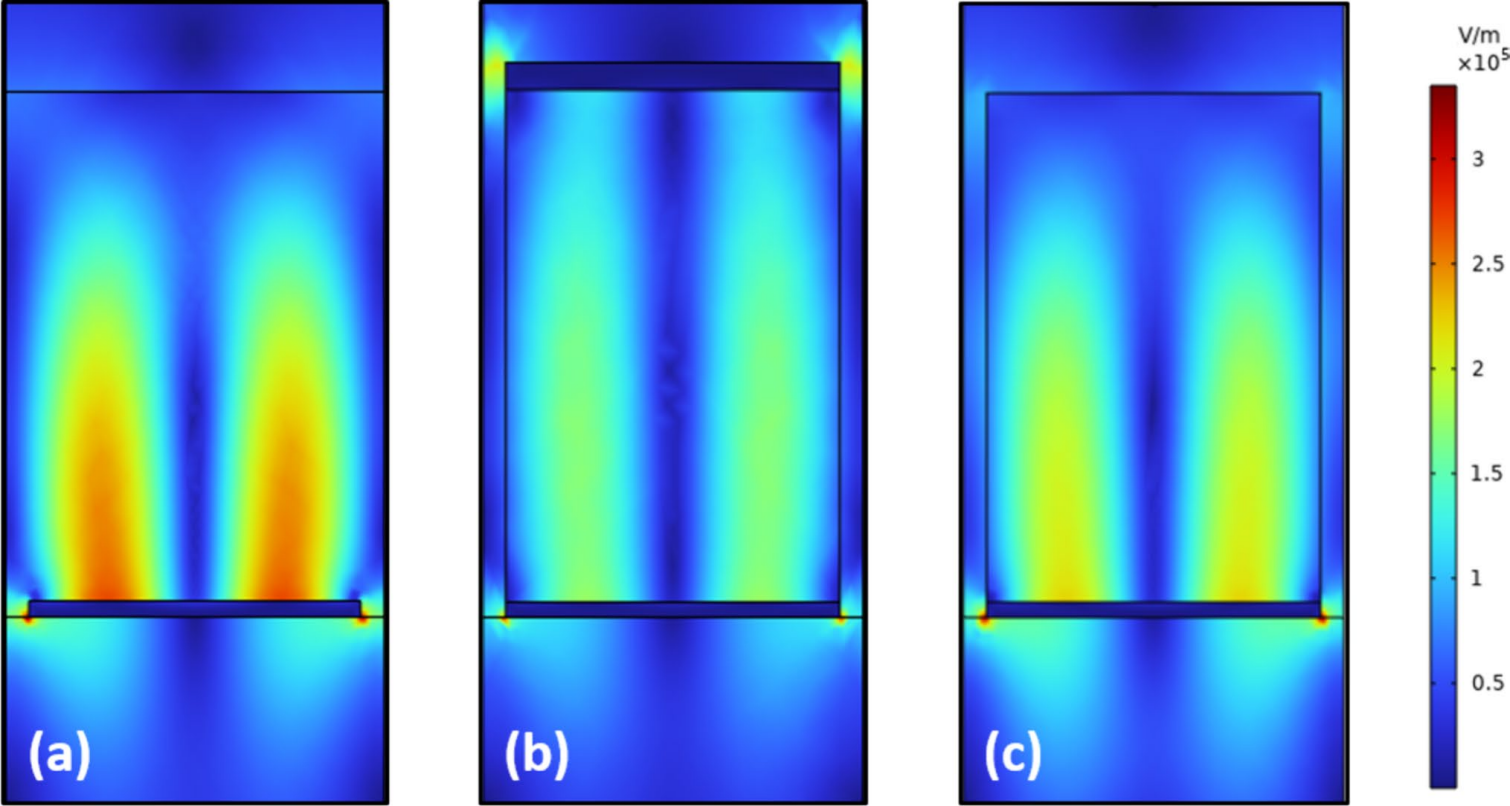
Distributions of electric field |*E*| in the structures at the resonant wavelength for **a** pGMR filter, **b** MIM-stack grating and **c** MI-stack grating

Based on a comprehension consideration of the filters performance across the whole band. The thickness of the bottom Au grating layer is fixed at *d*_a_ = 40 nm with a slit width of *w* = 120 nm, and the thickness of the stacks is set to *h* = 1.35 µm, with optimization methodologies gleaned from prior study [22]. Figure 2a–c shows the distributions of electric field |*E*| at resonant wavelength of 1500 nm in the unit cells of pGMR filter, MIM-stack grating and MI-stack grating, respectively. Locally enhanced electric field is observed at the corners of Au strips and shows strong resonance in the dielectric layer which are ascribed to the excitation of surface plasmon resonances (SPRs) and the phase-matched GMRs supported by the stack structures.

**Fig. 3:**
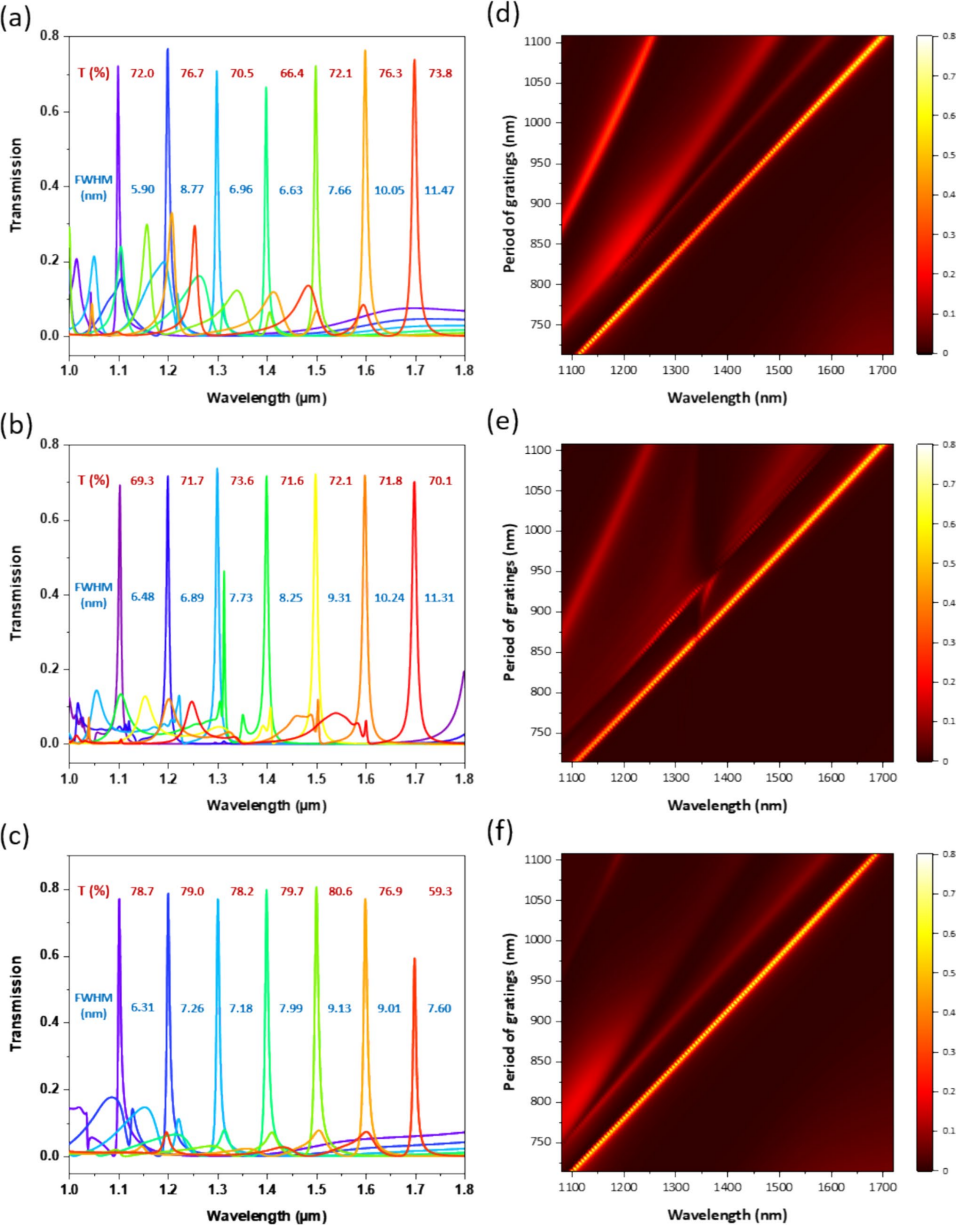
**a**–**c** Simulated transmission spectra and **d**–**f** two-dimensional transmittance plot of pGMR filters, MIM-stack gratings and MI-stack gratings with varying grating period. Under the specific condition of *d*_a_ = 40 nm, *w* = 120 nm, and *h* = 1.35 µm

We evaluate filter performance at wavelengths of 1100, 1200, 1300, 1400, 1500, 1600, and 1700 nm, and the calculated transmission spectra of the pGMR filters are depicted in Fig. 3a. By simply adjusting the Au grating period, narrowband transmission across a broad spectrum range can be achieved, with peak transmission ranging from 66.4 to 76.7% and FWHM spanning from 5.9 to 11.5 nm. Nonetheless, this structure also creates non-negligible sideband transmission at shorter wavelengths to the target transmission peak. Figure 3d illustrates the two-dimensional plot of transmission spectra for pGMR filters with varying grating period, wherein transmission bands disperse linearly with the grating period. Beside the target transmission peak, two sidebands can be seen clearly which may compromise filter accuracy during wide-spectrum analysis.

For MIM-stack grating structures, an Au capping layer is positioned atop the grating ridges, forming plasmonic resonators as shown in Fig. 1b. The Au-SU8-Au stack are separated by 120 nm slits with *d*_a_ = 40 nm, *h* = 1.35 µm, and the thickness of capping Au is optimized to *d*_a2_ = 70 nm. As depicted in Fig. 3b, the MIM-stack gratings exhibit stable narrowband transmission of around 70% at the target wavelengths and effective mitigation of sidebands. Figure 3e showcases substantial contrast between the main peak and other sidebands. However, it is noteworthy that for structures with grating periods of 850–925 nm, a noticeable sideband emerges at shorter wavelengths, accompanied by a degradation of the main peak in the vicinity of 1340 nm, possibly attributed to undesirable plasmon excitation at the top Au layer.

**Fig. 4:**
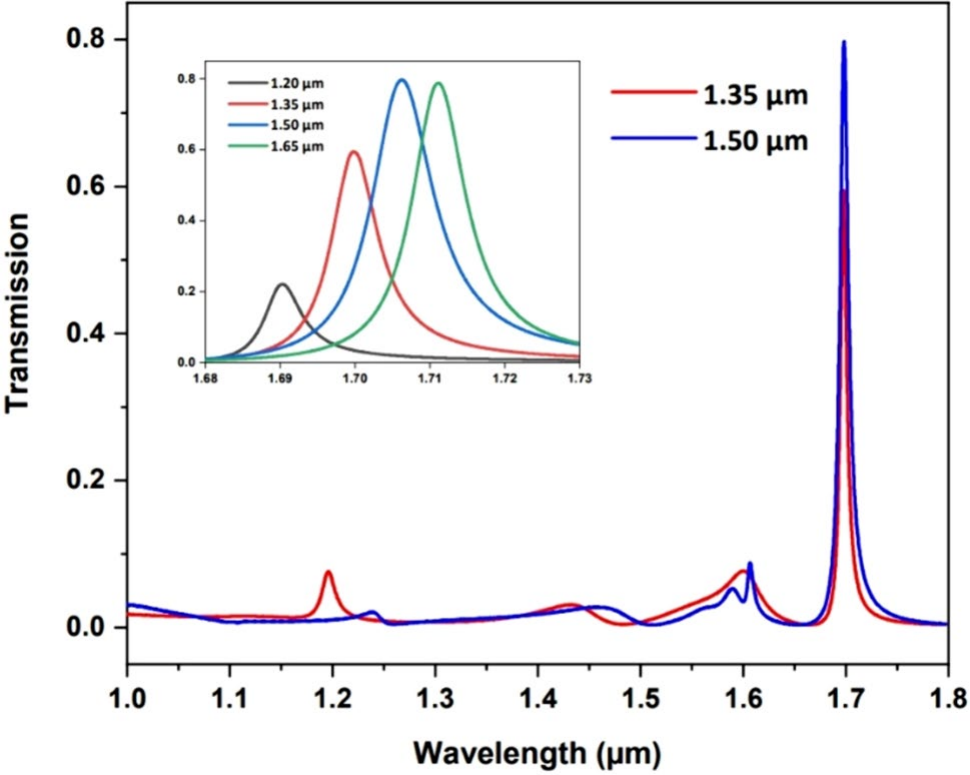
Simulated transmission spectra of MI-stack gratings operating at 1700 nm with *h* = 1.35 μm and 1.5 μm. Inset depicts the transmission performance of MI-stack gratings with varying dielectric thickness

The structure of MI-stack grating comprises an Au-SU8 stack atop the quartz substrate as shown in Fig. 1c. As illustrated in Fig. 3c, f, the sidebands are noticeably suppressed, potentially owing to the ulteriorly confined guided mode. The filters across the SWIR band attain a high transmittance of approximately 80% and demonstrate a consistency, except for the peak at 1700 nm where the transmittance reaches only 60%. To enhance filter efficiency at longer wavelengths, we investigate the effect of dielectric layer thickness on the filter performance. For comparative analysis, the calculated transmission spectra of filters with varying dielectric thicknesses (*h* ranging from 1.2 to 1.65 μm) are depicted in the inset of Fig. 4. As the dielectric thickness increases, there is an associated enhancement in peak transmission along with a red shift, reaching a maximum when h = 1.5 μm. By adjusting the grating period of the structure with a 1.5 µm SU8 dielectric layer, the filter operating at 1700 nm achieves a peak transmission as high as 80% with further mitigated sidebands. This observation suggests that a gradient dielectric thickness can be strategically employed to achieve optimized performance for filters operating at varying wavelengths.

The filters based on subwavelength gratings are achievable with state-of-the-art nanofabrication approaches. Subwavelength gold gratings on the quartz substrate can be fabricated through electron beam lithography (EBL) patterning and thermal evaporation deposition. In the case of the p-GMR structure, a continuous 1.35 µm thick SU8 photoresist thin film can be deposited using a modified coat process. For MI-stack and MIM-stack gratings, the grating structures can be realized by sequentially depositing the Au layers and the SU8 layer onto the quartz substrate, followed by patterning through focused ion beam (FIB). It is noteworthy that high-efficiency methods, such as nanoimprint lithography, can be further developed for the fabrication of large-scale filter arrays.

Since gratings are sensitive to the angle of incident light, the transmission properties of subwavelength grating structures are angle-sensitive. The peak position and intensity of the resonance, both for the main peak and the side bands, will shift with varying angles of incidence. Therefore, careful consideration of the angle of incidence is necessary for the practical application of these filters. Moreover, high index contrast dielectric gratings and/or two-dimensional grating design can be exploited for angle-tolerant and polarization-independent solutions.

## Conclusion

In this study, we explore the filtering efficacy of various subwavelength Au grating structures incorporating with dielectric SU8 components. The investigated structures, including pGMR filters, MIM-stack gratings, and MI-stack gratings, exhibit remarkable wavelength selectivity across a wide range spanning from 1100 to 1700 nm, with narrowband transmission characteristics. From a practical standpoint, pGMR filters offer advantages in terms of processing simplicity and scalability for large-scale fabrication. However, their filtering accuracy is compromised by the presence of sidebands generated at shorter wavelengths. In comparison, the utilization of an MIM-stack grating structure achieves an average transmission of 70% and mitigated sidebands. MI-stack grating filters demonstrate peak transmissions of approximately 80% with observable suppression of sidebands. Despite some degradation observed at longer wavelengths, MI-stack grating filters hold promise for further optimization through the employment of gradient dielectric layers. In summary, subwavelength gratings exhibit significant potential for narrowband filtering within the SWIR waveband. Moreover, their streamlined design offers the potential for large-scale fabrication of multiband filter arrays which can be compactly integrated with SWIR detector arrays.

## Data Availability

The data presented in this study are available on request from the corresponding author.
